# Predictors of outcomes in adults with acute myeloid leukemia and *KMT2A* rearrangements

**DOI:** 10.1038/s41408-021-00557-6

**Published:** 2021-09-29

**Authors:** Ghayas C. Issa, Jabra Zarka, Koji Sasaki, Wei Qiao, Daewoo Pak, Jing Ning, Nicholas J. Short, Fadi Haddad, Zhenya Tang, Keyur P. Patel, Branko Cuglievan, Naval Daver, Courtney D. DiNardo, Elias Jabbour, Tapan Kadia, Gautam Borthakur, Guillermo Garcia-Manero, Marina Konopleva, Michael Andreeff, Hagop M. Kantarjian, Farhad Ravandi

**Affiliations:** 1 Department of Leukemia, The University of Texas MD Anderson Cancer Center, TX Houston, USA; 2 Division of General Internal Medicine, University of Pittsburgh School of Medicine, PA Pittsburgh, USA; 3 Department of Biostatistics, The University of Texas MD Anderson Cancer Center, TX Houston, USA; 4 Division of Data Science, Yonsei University, Wonju, South Korea; 5 Department of Hematopathology, The University of Texas MD Anderson Cancer Center, TX Houston, USA; 6 Department of Pediatrics, The University of Texas MD Anderson Cancer Center, TX Houston, USA

**Keywords:** Leukaemia, Leukaemia

## Abstract

Acute myeloid leukemia (AML) with rearrangement of the *lysine methyltransferase 2a* gene (*KMT2Ar*) has adverse outcomes. However, reports on the prognostic impact of various translocations causing *KMT2Ar* are conflicting. Less is known about associated mutations and their prognostic impact. In a retrospective analysis, we identified 172 adult patients with *KMT2Ar* AML and compared them to 522 age-matched patients with diploid AML. *KMT2Ar* AML had fewer mutations, most commonly affecting *RAS* and *FLT3* without significant impact on prognosis, except for patients with ≥2 mutations with lower overall survival (OS). *KMT2Ar* AML had worse outcomes compared with diploid AML when newly diagnosed and at relapse, especially following second salvage (median OS of 2.4 vs 4.8 months, *P* < 0.0001). Therapy-related *KMT2A*r AML (t-AML) had worse outcomes compared with de novo *KMT2Ar* AML (median OS of 0.7 years vs 1.4 years, *P* < 0.0001). Allogeneic hematopoietic stem cell transplant (allo-HSCT) in first remission was associated with improved OS (5-year, 52 vs 14% for no allo-HSCT, *P* < 0.0001). In a multivariate analysis, translocation subtypes causing *KMT2Ar* did not predict survival, unlike age and allo-HSCT. In conclusion, *KMT2Ar* was associated with adverse outcomes regardless of translocation subtype. Therefore, AML risk stratification guidelines should include all *KMT2Ar* as adverse.

## Introduction

Chromosomal translocations involving 11q23 where the *lysine methyltransferase 2a* gene (*KMT2A)* is located cause acute leukemias with high rates of resistance and relapse following standard treatments [1]. Despite an increased understanding of the leukemogenic mechanisms caused by *KMT2A* (also known as *MLL*) rearrangements (*KMT2Ar*), less is known about determinants of response and resistance to current treatments. *KMT2Ar* leukemias affect the myeloid lineage, lymphoid lineage, or both. They are associated with an adverse prognosis when occurring in infants, children, or adults with leukemia [2–4]. The *KMT2A-MLLT3* fusion caused by t(9;11)(p21.3;q23.3) is the most common *KMT2Ar* in adults with AML, but more than 80 different fusion partners have been described [5].

Among chemotherapies associated with therapy-related AML (t-AML), topoisomerase II inhibitors are strongly associated with *KMT2Ar* leukemias, with a short latency to clinical manifestation [6]. In other genotypes of AML, previous exposure to chemotherapy leads to worse clinical outcomes, however, there have been conflicting reports on the prognostic impact of this factor in *KMT2Ar* AML [7–9]. The European LeukemiaNet (ELN) classifies t(9;11) as an intermediate-risk abnormality whereas other *KMT2Ar* AML were classified as adverse [10]. Nonetheless, results across studies on this difference in prognostic impact are not consistent, with some indicating similar adverse outcomes with all *KMT2Ar* AML [8, 9, 11, 12]. Genomic characterization of AML has refined prognostic models, although less is known about the mutational landscape of *KMT2Ar* AML and their prognostic impact [13]. Finally, much less is known about the clinical outcomes associated with *KMT2Ar* in the relapsed or refractory (R/R) setting.

We conducted a retrospective analysis of patients with *KMT2Ar* AML treated at our institution to characterize their genomic and phenotypic characteristics further and determine the association of these variables with prognosis and response to various lines of therapy.

## Methods

### Patient selection

We screened adult patients with AML treated at The University of Texas MD Anderson Cancer Center between January 1990 and December 2019. We identified 9465 patients with newly diagnosed AML, of whom 172 (2%) had *KMT2Ar*. We excluded patients with 11q23 translocations in whom *KMT2Ar* was not detected by fluorescence in situ hybridization (FISH) (Supplemental Fig. 1). Given that *KMT2Ar* occur in younger patients with AML, a cohort of patients with diploid karyotype were age-matched at a 3 to 1 ratio using propensity score and were used as a comparator for all subsequent analyses throughout this manuscript [14]. Among patients with *KMT2Ar*, 117 (68%) had mutational analysis done with targeted next-generation sequencing (NGS) panels. These panels included genes frequently involved in hematologic malignancies (panels of either 28, 53, or 81 genes depending on the time period; Supplemental Table 1) [15]. Fifty patients with *KMT2Ar* AML presenting to our institution at the time of relapse were included in the analysis of response and outcomes with subsequent lines of therapy (Supplemental Tables 2, 3). Treatment consisted of either high- or low-intensity regimens based on age and comorbidities. High-intensity regimens included combinations of cytarabine and idarubicin with or without a second nucleoside analog (i.e., cladribine, fludarabine, or clofarabine). Low-intensity regimens included either hypomethylating agents (i.e., azacitidine or decitabine) or low-dose cytarabine, with the addition of venetoclax more recently (starting in 2018) (Supplemental Table 4). Targeted therapies were added when available and indicated (Supplemental Table 4). Measurable residual disease (MRD) assessment was performed on bone marrow samples using multicolor flow cytometry as previously described [16]. This study was performed in accordance with the Declaration of Helsinki and was approved by the institutional review board.

### Statistical methods

Patient characteristics were summarized using medians and ranges for continuous variables and frequencies or percentages for categorical variables. Continuous variables were compared using the Wilcoxon rank-sum test for pairwise comparisons and the Kruskal–Wallis for multiple comparisons. Categorical variables were compared using Fisher’s exact test. Responses were defined according to the International Working Group recommendations [17]. Overall survival (OS) was calculated from the time of diagnosis in the newly diagnosed population or treatment start date in patients with relapsed disease, to the time of death or last follow-up. Cumulative incidence of relapse (CIR) was calculated from the time of complete response (CR) or CR with incomplete count recovery (CRi) until relapse, censored for death in morphological remission or if the patient was alive at last follow-up. To minimize potential lead-time bias, landmark analyses were used while assessing the impact of allogeneic hematopoietic stem cell transplant (allo-HSCT), where only patients in first remission lasting beyond the median time to transplant were included [18]. The Kaplan–Meier method was used to estimate the probability of OS or CIR and were compared by the log-rank test. Univariate and multivariate Cox proportional hazards models were used to assess the association between patient characteristics and outcomes. Allo-HSCT was included as a time-dependent variable, and variables with *P* ≤ 0.05 in the univariate analysis were included in the initial multivariate analysis. Backward model selection was used to eliminate variables until all remaining were statistically significant with *P* < 0.05. Analyses were performed using GraphPad Prism (San Diego, CA, USA) and SAS version 9.4 (Cary, NC, USA).

## Results

### Patient characteristics and *KMT2A* rearrangements

The median age at diagnosis of patients with *KMT2Ar* AML was 52 years (range, 17–85 years), with a relatively higher proportion of females (61 vs 49% in diploid AML, *P* = 0.01) (Table 1). As previously described, *KMT2Ar* AML commonly manifested as monocytic in 67% of patients compared with 33% in the diploid karyotype group (*P* < 0.0001), was associated with markers of proliferation such as a higher percentage of bone marrow blasts at diagnosis and a lower platelet count. In this cohort, 69 patients (40%) had t-AML and *KMT2Ar*.

**Table 1:** Baseline characteristics of newly diagnosed adults with AML.

Characteristic	*KMT2Ar*	Age-matched diploid	*P*
Patients, no.	172	522	
Median age, years (range)	52 (17–85)	52 (10–86)	
Female, no. (%)	104 (61)	256 (49)	0.01
Monocytic phenotype, no. (%)	116 (67)	170 (33)	<0.0001
WBC, median x 10^9^/L (range)	9.1 (1–270)	10.9 (0–390)	0.3
Platelets, median x 10^9^/L (range)	49 (3–279)	55 (1–635)	0.009
BM blast %, median (range)	76 (20–98)	52 (10–99)	<0.0001
t-AML, no. (%)	69 (40)	35 (7)	<0.0001
High-intensity treatment, no. (%)	145 (84)	432 (83)	0.6
Low-intensity treatment, no. (%)	27 (16)	90 (17)	
Allo-HSCT, no. (%)	46 (27)	118 (23)	0.3

High-intensity treatment includes the combination of cytarabine and idarubicin or the addition of a nucleoside analog to the combination. Low-intensity treatment includes treatment with hypomethylating agents, low-dose cytarabine, or targeted therapies.

*WBC* white blood cell, *BM* bone marrow, *LDH* lactate dehydrogenase, *Allo-HSCT* allogeneic hematopoietic stem cell transplant, *t-AML* therapy-related AML.

*P*: Kruskal–Wallis or Fisher exact test.

**Fig. 1:**
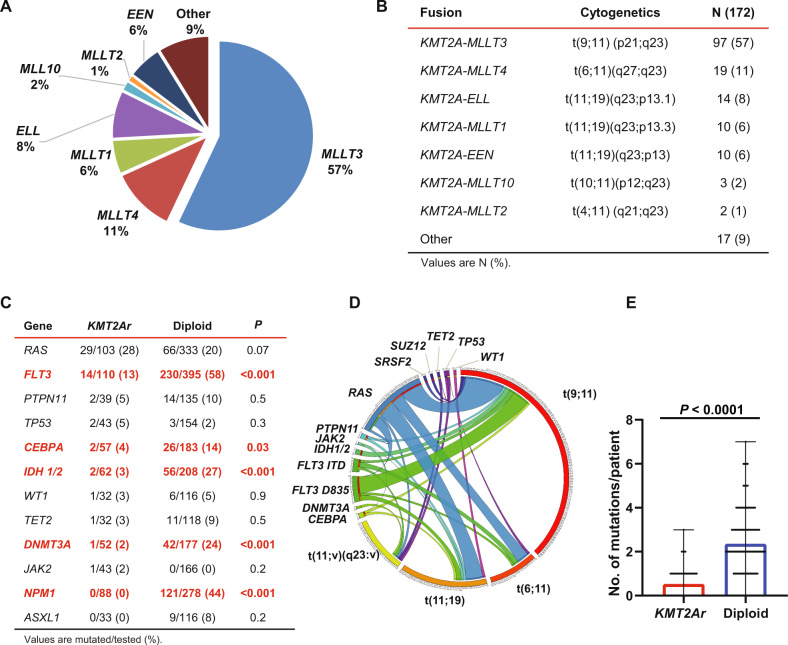
Fusion partner genes and mutational profile of adults with newly diagnosed *KMT2Ar* AML. **A** Distribution of fusion partner genes. **B** Cytogenetics and distribution of 11q23 translocations. **C** Genes most commonly mutated in *KMT2Ar* AML compared to an age-matched cohort of AML with a diploid karyotype. **D** Circos plot depicting patterns of co-occurrence between mutations and various translocations leading to *KMT2Ar*. **E** Number of mutations per patient comparing *KMT2Ar* AML to an age-matched cohort of AML with a diploid karyotype.

The most common translocations were t(9;11)(p21;q23)/*KMT2A-MLLT3* detected in 97 patients (57%), followed by t(6;11)(q27;q23)/*KMT2A-MLLT4* detected in 19 patients (11%), t(11;19)(q23;p13.1)/*KMT2A-ELL* in 14 patients (8%), t(11;19)(q23;p13.3)/*KMT2A-MLLT1* in 10 patients (6%), t(11;19)(p23;q13)/*KMT2A-EEN* in 10 patients (6%), t(10;11)(q12;q23)/*KMT2A-MLLT10* in 3 patients (2%), and t(4;11)(q21;q23)/*KMT2A-MLLT2* in 2 patients (1%) (Fig. 1A, B). Seventeen patients (9%) had less common translocations occurring in ≤2 patients (Supplemental Table 5). Baseline characteristics by 11q23 translocations were mostly similar (Supplemental Table 6). Notably, t(9;11) presented more commonly with monocytic features compared to t(11:19) (75 vs 56%, *P* = 0.048) and t(11;v)(q23:v) (75 vs 45%, *P* = 0.009). No other significant differences in the characteristics were found, including rates of allo-HSCT.

### Mutational profile and immunophenotype

Among patients with newly diagnosed AML, 117 patients (68%) had targeted NGS assessment of their diagnostic bone marrow specimens (Supplemental Fig. 2). Most patients examined had no additional mutations detected (65 of 117 patients or 56%). Overall, the analysis yielded 63 mutations in 52 patients with ≥1 mutation (52 of 117 patients or 44%). The median number of mutations per patient in the *KMT2Ar* cohort was 0.5 mutation (range, 0–3), which was significantly lower compared to diploid AML (median of two mutations/patient, range 0–7, *P* < 0.0001) (Fig. 1E). The most common mutations in *KMT2Ar* AML involved *RAS* in 29 of 103 patients (28%), followed by *FLT3* in 14 of 110 patients (13%). These mutations were predominately D835 *FLT3* kinase domain mutations (nine patients, 8%), and less commonly *FLT3* internal tandem duplications (five patients, 5%). Mutations in *FLT3* and *RAS* co-occurred in 5 of 110 patients (5%) (Fig. 1D and Supplemental Fig. 2). In addition, *KMT2Ar* patients had mutations in *PTPN11* (5%), *TP53* (5%), *CEBPA* (4%), and *IDH1* (3%). Therefore, mutations associated with *KMT2Ar* most commonly involved the *RAS* pathway (*RAS* and *PTPN11*, with one patient having co-occurrence of these mutations).

The mutational landscape of *KMT2Ar* AML significantly differed from that of diploid AML (Fig. 1C). Mutations usually seen in clonal hematopoiesis were rare in *KMT2Ar* AML [19–21]. A *DNMT3A* mutation was detected in 1 of 52 patients (2 vs 24% in diploid karyotype, *P* < 0.001), a *TET2* mutation in 1 of 32 patients (3 vs 9% in diploid karyotype, *P* = 0.5), and no *ASXL1* mutations were detected (vs 8% in diploid karyotype, *P* = 0.2). Furthermore, there were no *NPM1* mutations among 88 patients with *KMT2Ar* AML, and mutations in *FLT3*, *IDH*, and *CEBPA* were significantly less common when compared to diploid AML (Fig. 1C and Supplemental Fig. 2). There was no difference in the distribution of these mutations among different 11q23 translocations (Fig. 1D and Supplemental Table 7).

We analyzed by flow cytometry the immunophenotype of patients with *KMT2Ar* AML compared with diploid AML. We found that *KMT2Ar* AML was characterized by lower expression of CD7 (median: 4 vs 14%, *P* < 0.001), CD13 (median: 58 vs 72%, *P* < 0.001), and CD34 (median: 16 vs 34%, *P* < 0.001), and higher expression of CD33 (median: 94 vs 85%, *P* < 0.001) (Supplemental Fig. 3).

### Morphologic and cytogenetic responses

Patients with newly diagnosed *KMT2Ar* AML had lower response rates to therapy compared with diploid AML with a CR/CRi rate of 72 vs 81% respectively (*P* = 0.01) (Table 2). However, the rate of MRD negative remission in evaluable patients who achieved a morphologic response was similar between the two groups (68 vs 68%, *P* = 0.9). Notably, patients with *KMT2Ar* had higher rates of early mortality compared to diploid AML with a 60-day mortality rate of 15 vs 7% (*P* = 0.004). There was no statistically significant difference in response rates when comparing various 11q23 translocations, although t(6;11) tended to have a lower CR rate compared to t(9;11) (58 vs 65%, *P* = 0.9) (Supplemental Table 8). As expected, the probability of achieving a response decreased with each line of therapy following relapse. The CR/CRi rates following first, second, or ≥3rd line therapies were 72, 43, and 9% respectively (Supplemental Table 9). However, *KMT2Ar* was associated with lower response rates at relapse, especially following ≥3rd line of therapy, where the CR/CRi rate was 9 vs 31% for diploid AML (*P* < 0.001) (Supplemental Table 9).

**Table 2:** Response and early mortality rates of newly diagnosed adults with AML.

Best Response	*KMT2Ar*	Age-matched diploid	*P*
N	172	522	
CR	113 (66)	403 (77)	0.01
CRi	10 (6)	18 (4)	
CR + CRi	123 (72)	421 (81)	
No response	49 (28)	101 (19)	
MRD negative by MFC (%)	17/25 (68)	90/132 (68)	0.9
*Early mortality rates*			
30-day mortality	17 (10)	20 (4)	0.005
60-day mortality	26 (15)	38 (7)	0.004

Values are *n* (%).

*CR* complete remission, *CRi* complete remission with incomplete hematologic recovery, *MRD* measurable residual disease assessed by multiparameter flow cytometry (MFC) following induction in evaluable patients.

**Fig. 2:**
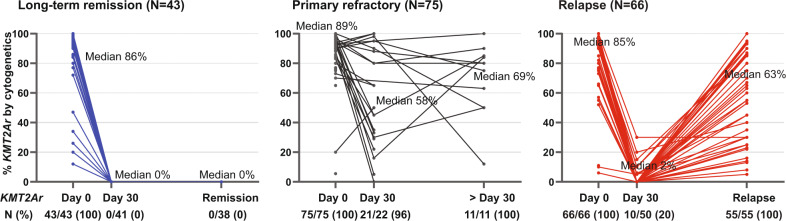
Dynamic changes of the cytogenetic burden in adults with *KMT2Ar* AML following treatment. Numbers depict the estimated % of *KMT2Ar* measured by fluorescence in situ hybridization (FISH) or conventional cytogenetics when FISH was not performed. Numbers below the X-axis indicate the proportion (%) of patients with *KMT2Ar* among those with available cytogenetic data. This analysis included unique patients from the newly diagnosed cohort in addition to patients who presented to our institution with relapsed or refractory disease. The long-term remission graph depicts those who achieved and maintained a morphologic remission whereas the relapse graph depicts those with initial morphologic remission following induction treatment and subsequent relapse.

To assess the validity of *KMT2Ar* as a marker of disease evolution, we tracked the cytogenetic burden following treatment in the frontline and the R/R cohorts (Fig. 2). All patients who achieved a CR/CRi and a sustained remission following treatment had no detectable *KMT2Ar* by conventional cytogenetics or FISH on monitoring (0 out of 38 patients). Importantly, none of the patients with long-term remission had *KMT2Ar* detected on assessments done following induction treatment around Day 30, therefore highlighting the importance of achieving early cytogenetic remission in this setting. Conversely, patients who achieved morphologic remission (CR/CRi) and subsequently relapsed had a higher proportion of *KMT2Ar* detected at Day 30 following induction (10 of 50 patients or 20%). At the time of relapse, all patients with a cytogenetic analysis done had persistence of the *KMT2Ar* (55 of 55 patients or 100%). This suggests that *KMT2Ar* are founding events in this leukemia and that relapse following current treatments is not driven by the acquisition of novel drivers in the absence of *KMT2Ar*.

### Relapse and overall survival

**Fig. 3:**
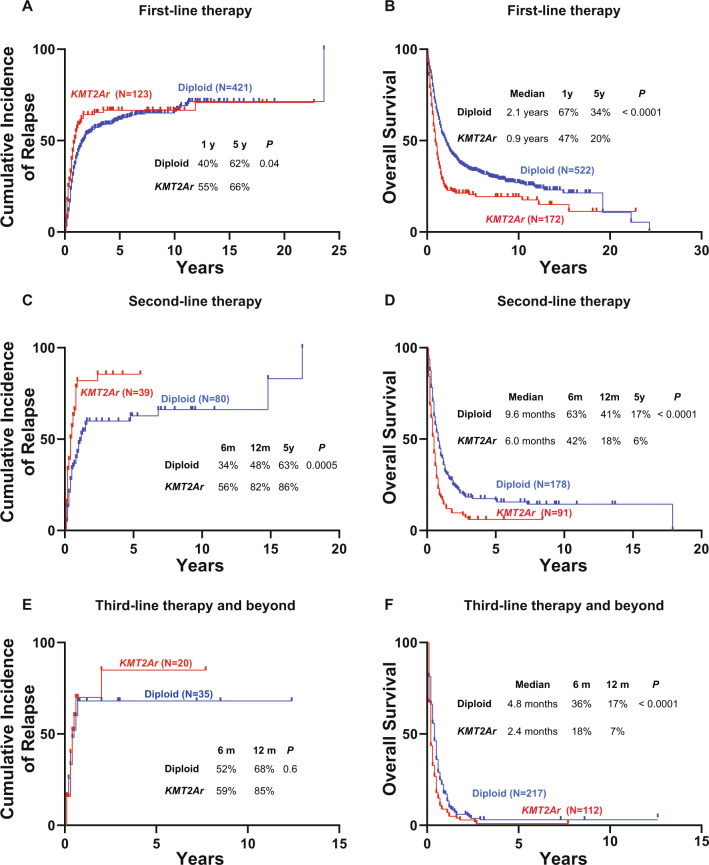
Cumulative incidence of relapse and overall survival for patients with *KMT2Ar* AML by a line of therapy compared with an age-matched cohort of AML with diploid karyotype. **A** Cumulative incidence of relapse following first-line therapy. **B** Overall survival following first-line therapy. **C** Cumulative incidence of relapse following second-line therapy. **D** Overall survival following second-line therapy. **E** Cumulative incidence of relapse following third-line therapy. **F** Overall survival following third-line therapy. Treatment start date for second-line treatment and beyond was used for the calculation of time-to-event.

The median follow-up for this cohort was 8.1 years. Patients with newly diagnosed *KMT2Ar* AML had a higher risk of relapse compared to diploid AML with a CIR at 5 years of 66 vs 62% (*P* = 0.04) (Fig. 3A). These patients also had a worse OS rate: median OS of 0.9 years vs 2.1 years for diploid patients and a 5-year OS of 20 vs 34% (*P* < 0.0001). Following the first relapse, patients with *KMT2Ar* AML had a higher risk of subsequent relapse and worse OS compared to diploid AML with each line of therapy (Fig. 3C, D, E, F). The median OS of patients with *KMT2Ar* AML following 1st, 2nd, and ≥3rd lines therapies was 10.8 months, 6 months, and 2.4 months, respectively compared with 2.1 years, 9.6 months, and 4.8 months for diploid AML (*P* < 0.0001) (Fig. 3B, D, F).

**Fig. 4:**
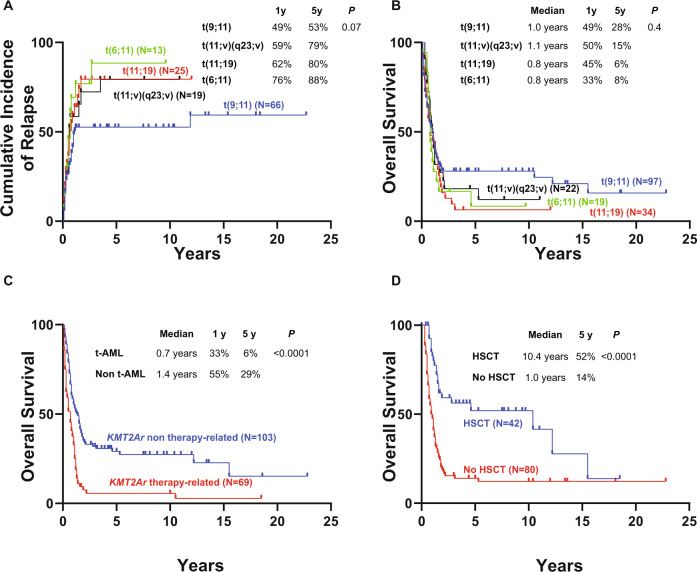
Risk of relapse and overall survival in newly diagnosed *KMT2Ar* AML. **A** Cumulative incidence of relapse by subtype of *KMT2Ar*. **B** Overall survival by subtype of *KMT2Ar*. **C** Overall survival of newly diagnosed *KMT2Ar* AML by therapy-related status**. D** Landmark analysis comparing overall survival of patients with newly diagnosed *KMT2Ar* AML who underwent an allogeneic hematopoietic stem cell transplant following the first remission to those who did not undergo transplant.

When newly diagnosed, including all patients with *KMT2Ar* AML regardless of age, t(9;11) was associated with slightly better outcomes compared to other *KMT2Ar* though these differences were not statistically significant (Fig. 4A, B). The median OS of patients with t(9;11) was 1 year with 5-year CIR and OS rates of 53 and 28%, respectively. t(6;11) was associated with the highest risk of relapse with a 5-year CIR of 88% and a median OS of 0.8 years and a 5-year OS of 8%. Similarly, patients with t(11;19) had a higher risk of relapse and worse OS with a median OS of 0.8 years and 5-year CIR and OS rates of 80 and 6%, respectively. Outcomes were better in younger patients (age <60 years) with newly diagnosed *KMT2Ar* AML, however, there was no statistically significant difference in OS comparing t(9;11) to other *KMT2Ar* (Supplemental Fig. 4C, D). However, patients with therapy-related *KMT2Ar* AML had a significantly higher chance of relapse and a lower likelihood of long-term survival compared to de novo *KMT2Ar* AML with a median OS of 0.7 years (vs 1.4 years, *P* < 0.0001) and 5-year CIR and OS rates of 80 (vs 65%, *P* = 0.009) and 6% (vs 29%, *P* < 0.0001), respectively (Fig. 4C and Supplemental Fig. 5). The difference in outcomes for the therapy-related disease was also noted in t(9;11) with a median OS of 0.5 years (vs 1.9 years, *P* < 0.0001) and 5-year CIR and OS rates of 62% (vs 48%, *P* = 0.1) and 9% (vs 48%, *P* < 0.0001) respectively (Supplemental Fig. 4A, B). There was no difference in outcomes comparing patients with *KMT2A* translocations and additional cytogenetic chromosomal abnormalities to those with *KMT2A* translocations only (Supplemental Figs. 6A, B and 7).

### Prognostic impact of additional mutations in *KMT2Ar* AML

Given that mutational analysis in AML has improved risk stratification by adding to the established value of cytogenetic abnormalities, we analyzed the impact of additional mutations on outcomes of *KMT2Ar* AML [13, 22]. Mutations in either *RAS* or *FLT3* did not affect OS in evaluable patients (Supplemental Fig. 8C, D). Compared to patients with *KMT2Ar* AML without detectable mutations, those who harbored any additional mutation also had a trend for worse OS though not statistically significant (5-year OS 14 vs 29% respectively, *P* = 0.1) (Supplemental Fig. 8A). However, a minority of patients who harbored ≥2 mutations (8 of 117 patients or 7%) had worse OS with a median of 0.5 years vs 1.1 years in those with fewer mutations (*P* = 0.01) (Supplemental Fig. 8B). These patients had co-occurrence of either *RAS*, *FLT3*, or *PTPN11* mutations (six of eight patients), *TET2* and *TP53* mutations in one patient, and *JAK2* and *CEBPA* in the remaining patient.

### HSCT in *KMT2Ar* AML

In this cohort, 46 patients (27%) with *KMT2Ar* AML received an allo-HSCT. Among them, 42 (24%) were transplanted in the first remission as consolidation. We performed a landmark analysis to compare outcomes of patients with *KMT2Ar* AML who had allo-HSCT in the first remission compared to those who did not. Allo-HSCT was associated with significantly improved OS with a median of 10.4 years (vs 1 year for evaluable non-transplanted patients) and 5-year OS of 52% (vs 14% for evaluable non-transplanted patients, *P* < 0.0001) (Fig. 4D). The median time to relapse for the remaining four patients who received an allo-HSCT as salvage therapy was 7.8 months with an OS of 0.7 years.

### CNS disease

*KMT2Ar* leukemias are frequently associated with central nervous system (CNS) involvement. Among newly diagnosed patients with *KMT2Ar* AML, 21 patients (12%) had CNS disease, three of whom had it at presentation (2%), whereas the rest developed it later in their treatment (17 patients or 10%). We next assessed the characteristics of these patients and whether there were predictors at baseline of CNS disease. When compared to *KMT2Ar* AML without CNS involvement (CNS-), we found no difference in variables usually thought to confer an increased risk of CNS involvement such as elevated white blood cell count, a monocytic phenotype (72 vs 67% in CNS- disease, *P* = 0.8) or presence of extramedullary involvement (35 vs 36% in CNS- disease, *P* = 0.8) (Supplemental Table 10). However, there was a higher proportion of patients with mutations in *FLT3* among those with CNS involvement, though not statistically significant (22 vs 11% in CNS−, *P* = 0.2). Among *KMT2Ar* patients with CNS involvement, 13 of 21 patients (62%) successfully cleared their spinal fluid from leukemia cells following intrathecal and systemic therapies. There was no statistically significant difference in OS between the *KMT2Ar* CNS + and CNS− groups, though CNS + patients had a trend for worse long-term survival (5-year OS 0 vs 23%, *P* = 0.09) (Supplemental Fig. 9).

### Predictors of relapse or death in *KMT2Ar* AML

**Fig. 5:**
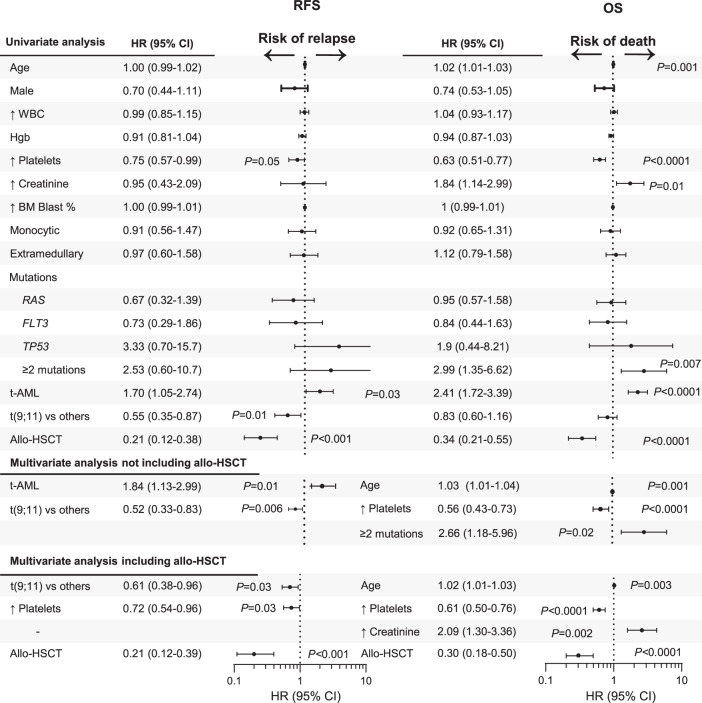
Univariate and multivariate analyses of factors predicting risks of relapse or death in newly diagnosed *KMT2Ar* AML. Variables with *P* ≤ 0.05 were included in the multivariate analysis. RFS relapse-free survival, OS overall survival, HR hazard ratio, WBC white blood cell count, Hgb hemoglobin, BM bone marrow, t-AML therapy-related AML, allo-HSCT allogeneic hematopoietic stem cell transplant.

In order to assess the impact of confounding variables on prognosis, we conducted univariate and multivariate analyses predicting risks of relapse or death in patients with *KMT2Ar* AML (Fig. 5). Allo-HSCT was identified as an independent factor significantly associated with a decreased risk of relapse with a hazard ratio (HR) of 0.21 (95% CI 0.12–0.39, *P* < 0.001) and decreased risk of death with an HR of 0.30 (95% CI 0.18–0.50, *P* < 0.0001) (Fig. 5). Despite a decreased risk of relapse, t(9;11) was not associated with improved risk of death compared to other *KMT2Ar*, even when the transplant was not included in the multivariate analysis, which was done in order to address the potential beneficial effect of allo-HSCT on all confounding adverse features. Therapy-related status in *KMT2Ar* AML independently increased the risk of relapse when the transplant was not considered in the multivariate analysis with an HR of 1.84 (95% CI 1.13–2.99, *P* = 0.01), however, this prognostic impact was abrogated by the addition of allo-HSCT to the model. Similarly, having ≥2 mutations in addition to *KMT2Ar* independently predicted for higher risk of death with an HR of 2.66 (95% CI, 1.18–5.96, *P* = 0.02), but was not a predictor when allo-HSCT was added to the model. The only other factors identified as independent predictors of the risk of death were age, low platelet count, and an elevated creatinine at diagnosis.

## Discussion

In this study, we show that the mutational landscape of *KMT2Ar* AML is unique and characterized by a relative paucity of mutations, where almost all cells at diagnosis have *KMT2Ar* by cytogenetic analysis, which persists following resistance or relapse. This confirms previous clinical observations and laboratory studies indicating that *KMT2Ar* is the founding event and a potent driver in this leukemia with a minimal contribution by additional mutations [23–28]. Mutations most commonly involved the *RAS* or *FLT3* genes and did not affect prognosis. These mutations are likely subclonal and occur later in the pathogenesis of the disease but possibly confer some proliferative advantage [29]. The rare occurrence of mutations frequently detected in clonal hematopoiesis indicates that *KMT2Ar* are not preceded by a precursor state, a common feature of non-fusion-driven AML [20]. However, a minority of patients had two or three mutations in addition to *KMT2Ar* which was associated with an adverse prognosis. These patients had co-occurrence of mutations in either *RAS*, *FLT3*, or *PTPN11*.

The incidence of *KMT2Ar* in this cohort of adults with newly diagnosed AML was 2% (172 of 9465 patients), which is lower than the incidence reported in other studies of 3–7% [3, 8, 9]. This is likely because we chose to focus our analysis on confirmed rearrangements when FISH was performed, while other studies have included other 11q23 abnormalities such as deletions. These 11q23 abnormalities without a clear translocation partner identified by conventional cytogenetics could harbor cryptic *KMT2Ar* translocations in up to 45% of cases in one estimate [30]. We identified 4 of 50 patients (8%) in the relapsed cohort with a cryptic *KMT2Ar*, while none were detected in the frontline cohort (Supplemental Fig. 10). In addition to routine FISH use at AML diagnosis, incorporation of a novel clinical assay based on RNA-sequencing or whole genome sequencing would improve the detection of cryptic *KMT2A* translocations [31–33]. Targeted sequencing performed in this cohort did not include identification of *KMT2A* partial tandem duplications, a well-described prognostic alteration involving this gene [34, 35].

We show that *KMT2Ar* AML in adults is characterized by high expression of CD33 (median expression of 94%), the target of gemtuzumab ozogamicin (GO). This is in line with a previous report on the immunophenotype of 17 of 19 *KMT2Ar* AML expressing CD33 (median expression 77%) [36]. This finding could bolster efforts investigating the addition of GO to the treatment of *KMT2Ar* AML in adults. A recent study by the Children’s Oncology Group demonstrated improved outcomes with the addition of GO in pediatric *KMT2Ar* AML [37]. As previously described, we found in this study a high rate of CNS involvement in adults with *KMT2Ar* AML (10 vs 3% in a general AML population). The NCCN (National Comprehensive Cancer Network) guidelines recommend a screening lumbar puncture (LP) to assess for CNS involvement in asymptomatic patients with monocytic AML, mixed phenotype acute leukemia, extramedullary disease, WBC >40 × 10^9^/L, or presence of *FLT3* mutations [38, 39]. Using these criteria, most adult patients with *KMT2Ar* AML would need a screening LP. We attempted to discriminate further predictors of CNS disease in *KMT2Ar* AML and found no clear predictors of CNS involvement in this entity.

There is a paucity of data on the outcomes of patients with relapsed *KMT2Ar* AML. This is the first study to our knowledge to examine outcomes for these patients with each line of therapy. In this study, patients with *KMT2Ar* AML had significantly worse outcomes following relapse compared to an age-matched population with a diploid karyotype. We found that the median OS for *KMT2Ar* AML after second-line therapy was 6 months and 2.4 months only after third-line therapy. This highlights the need to improve outcomes for patients with *KMT2Ar* AML, and the results of this study could be used when comparing available treatments to promising novel therapies in clinical development. Notably, early results from clinical trials investigating menin inhibitors, which are novel targeted agents for *KMT2Ar* leukemias or leukemias with other susceptible genotypes are highly encouraging [40–42].

A recent study by the Cancer and Leukemia Group B (CALGB) found that outcomes of patients with de novo AML, t(9;11) and age <60 years, excluding those who underwent allo-HSCT in first CR, were comparatively better than those with other 11q23 translocations (108 patients in this analysis) [25]. This was consistent with previous studies by the German Acute Myeloid Leukemia Intergroup where a cohort of 180 patients with 11q23 aberrations and age <60 years, were analyzed and t(9;11) had comparatively better outcomes [9]. We show similar results in our cohort when applying the same analysis criteria by restricting it to age <60 years, and de novo AML (Supplemental Fig. 4E). However, patients with these characteristics form only 26% (44 of 172 patients in our cohort) of adult patients with newly diagnosed *KMT2Ar* AML. Therefore, these results should not be applied to the general population with *KMT2Ar* AML. This study shows that the prognosis of *KMT2Ar* in adults with AML is adverse, regardless of the translocation, when looking at a diverse (all ages, de novo, and t-AML) large cohort of patients. Despite a lower risk of relapse in t(9;11) compared to other *KMT2Ar*, the difference in OS was not significant albeit a trend and was not an independent predictor of survival in the multivariate analysis. Therefore, risk stratification criteria such as the ones by the NCCN or the ELN, should reflect this data when indicating that among *KMT2A* rearrangements, t(9;11) is in the intermediate-risk category [38, 43]. The intermediate-risk assignment should apply only to patients with age <60 years, and non-therapy related t(9;11). Or alternatively, revise the criteria to include all *KMT2Ar* in the adverse risk category which would better reflect the characteristics of patients seen in the clinic. This is critical as we have shown that allo-HSCT in first CR is associated with significantly improved outcomes in all *KMT2Ar* AML. Certainly, no prognostic model is perfect, however, these risk models are used in clinical practice to determine which patients should undergo a consolidative allo-HSCT. Including all *KMT2Ar* in the adverse risk group would emphasize the importance of transplant for these patients. Current risk stratification models were developed largely based on cohorts of younger, fit patients with de novo AML, treated with intensive chemotherapy despite the fact that AML is largely a disease of older age (median age at diagnosis of 68). The advent of highly effective venetoclax-based therapies for older patients with AML invites for more inclusive risk stratification models regardless of age [44].

In conclusion, *KMT2Ar* is associated with adverse outcomes in adults with AML. It has a low mutational burden with a minimal associated prognostic impact. Adverse risk in *KMT2Ar* AML is worst in older patients and those with t-AML. Consolidation with an allo-HSCT following the first remission is associated with significantly improved outcomes leading to long-term survival. Outcomes of patients with relapsed *KMT2Ar* AML are dismal, highlighting the need for novel therapeutic strategies.

## Supplementary information

Supplement
