# Supplementary material for: Predictors of outcomes in adults with acute myeloid leukemia and KMT2A rearrangements
Source: Blood Cancer J. 2021 Sep 29;11(9):162. doi: 10.1038/s41408-021-00557-6 (PMC8481264; doi:10.1038/s41408-021-00557-6)
Supplement: Supplementary file 1 — Supplement [file 41408_2021_557_MOESM1_ESM.pdf]

Supplemental Figure 1: Patient selection.

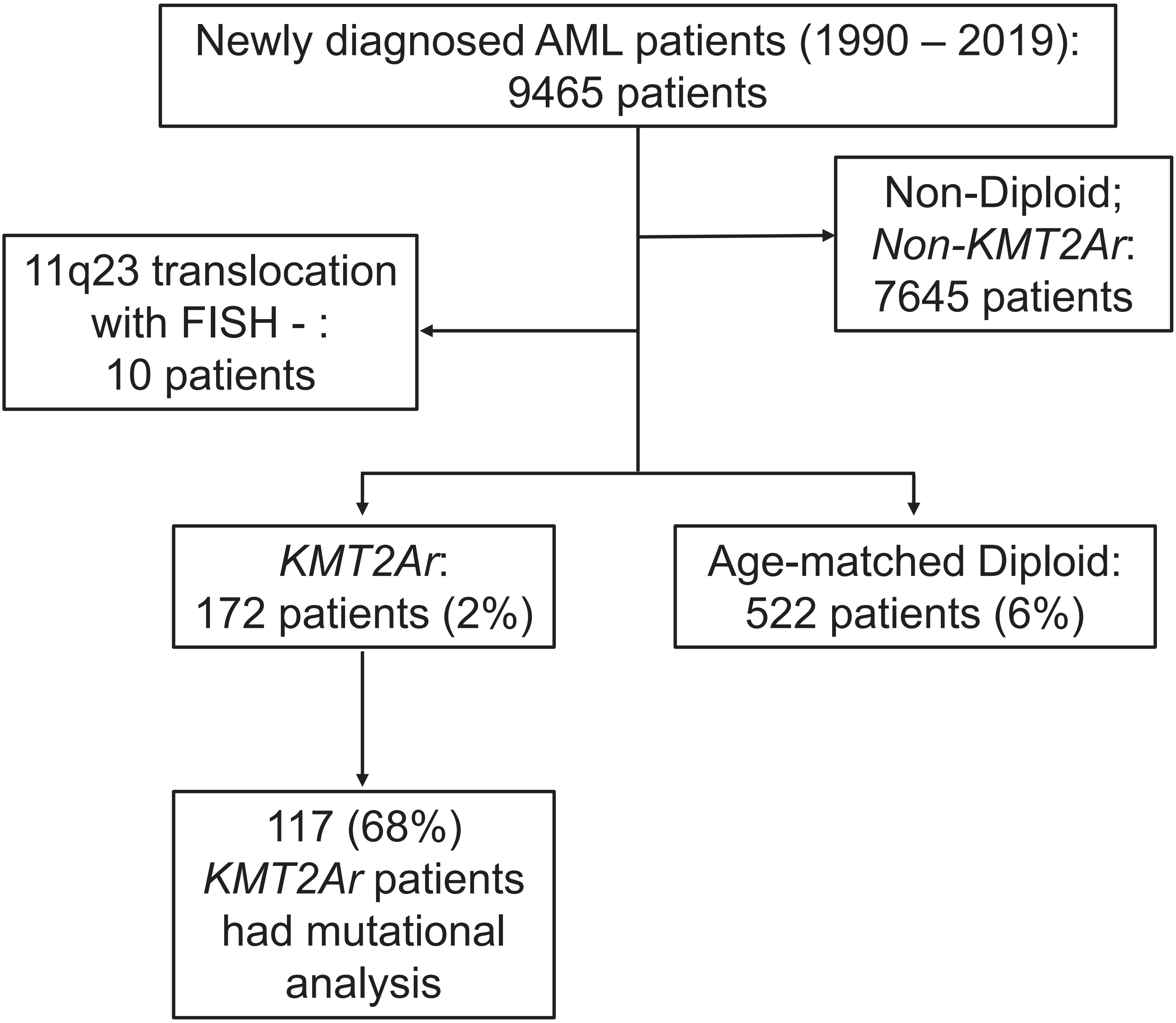

Supplemental Figure 2: Distribution of mutations per patient and by type of 11q23 translocation.

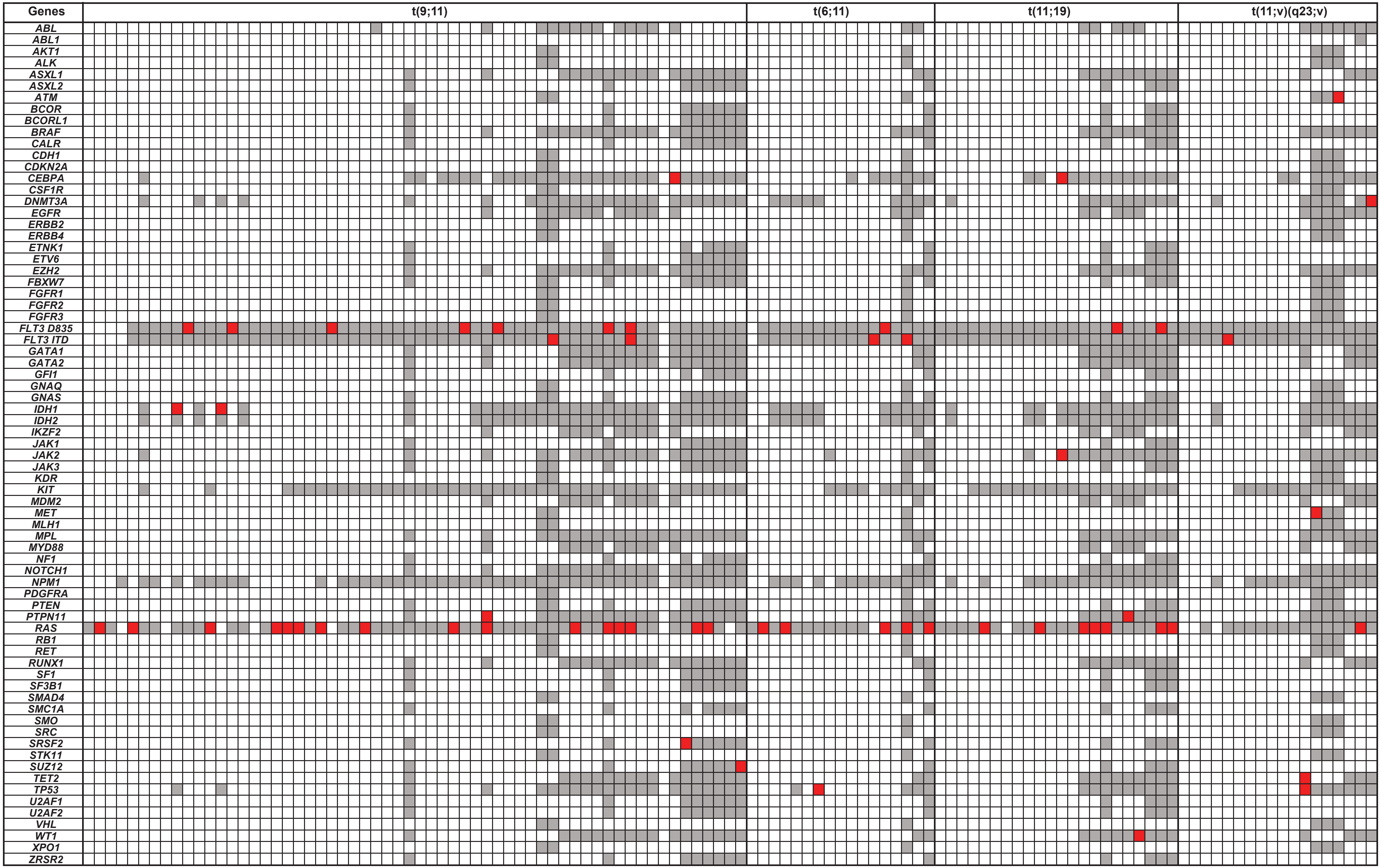

Supplemental figure 2:
Oncoprint of mutations detected in 117 patients with *KMT2Ar* AML.
Each column represents a patient and each row represents a single gene. Red, mutated; Grey, wildtype; Blank, mutational analysis not done.

Supplemental Figure 3: Immunophenotype of *KMT2Ar* compared to diploid karyotype AML.

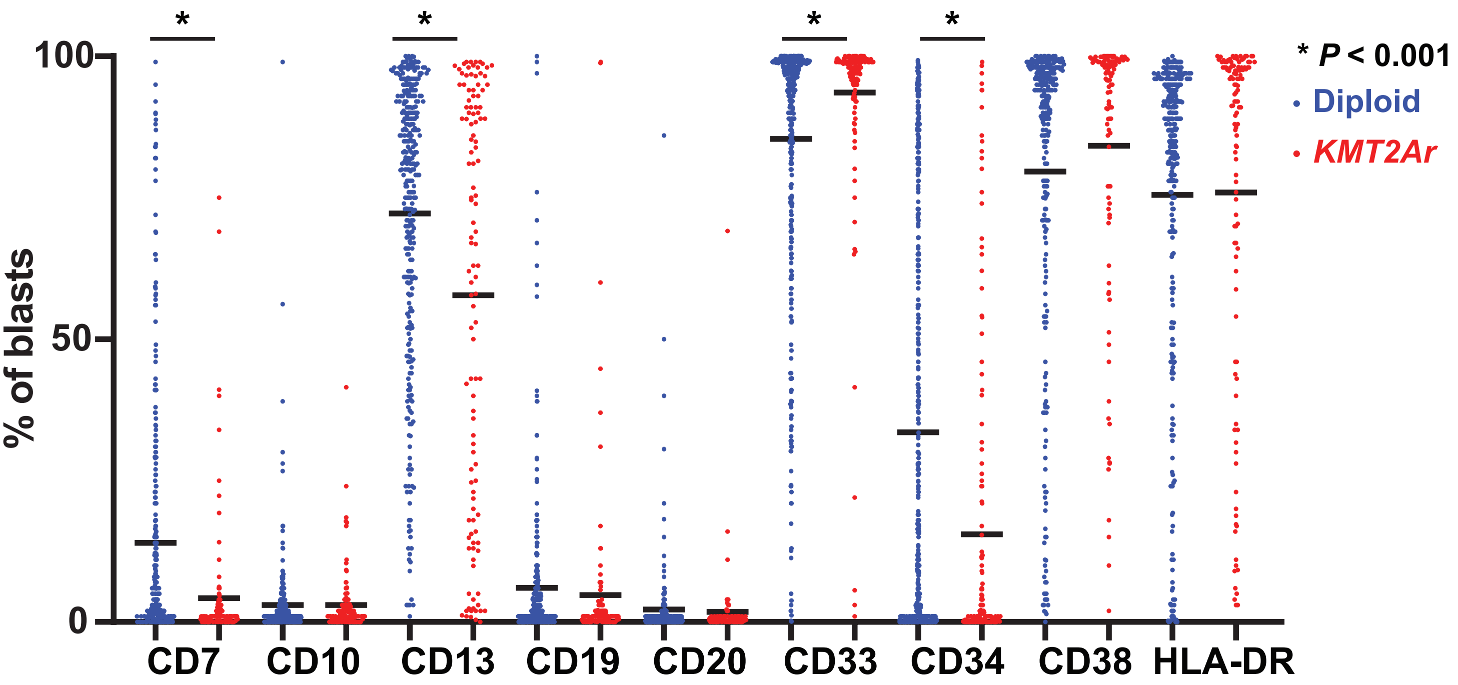

Supplemental Figure 4: Risk of relapse and overall survival of patients with t(9;11) AML.

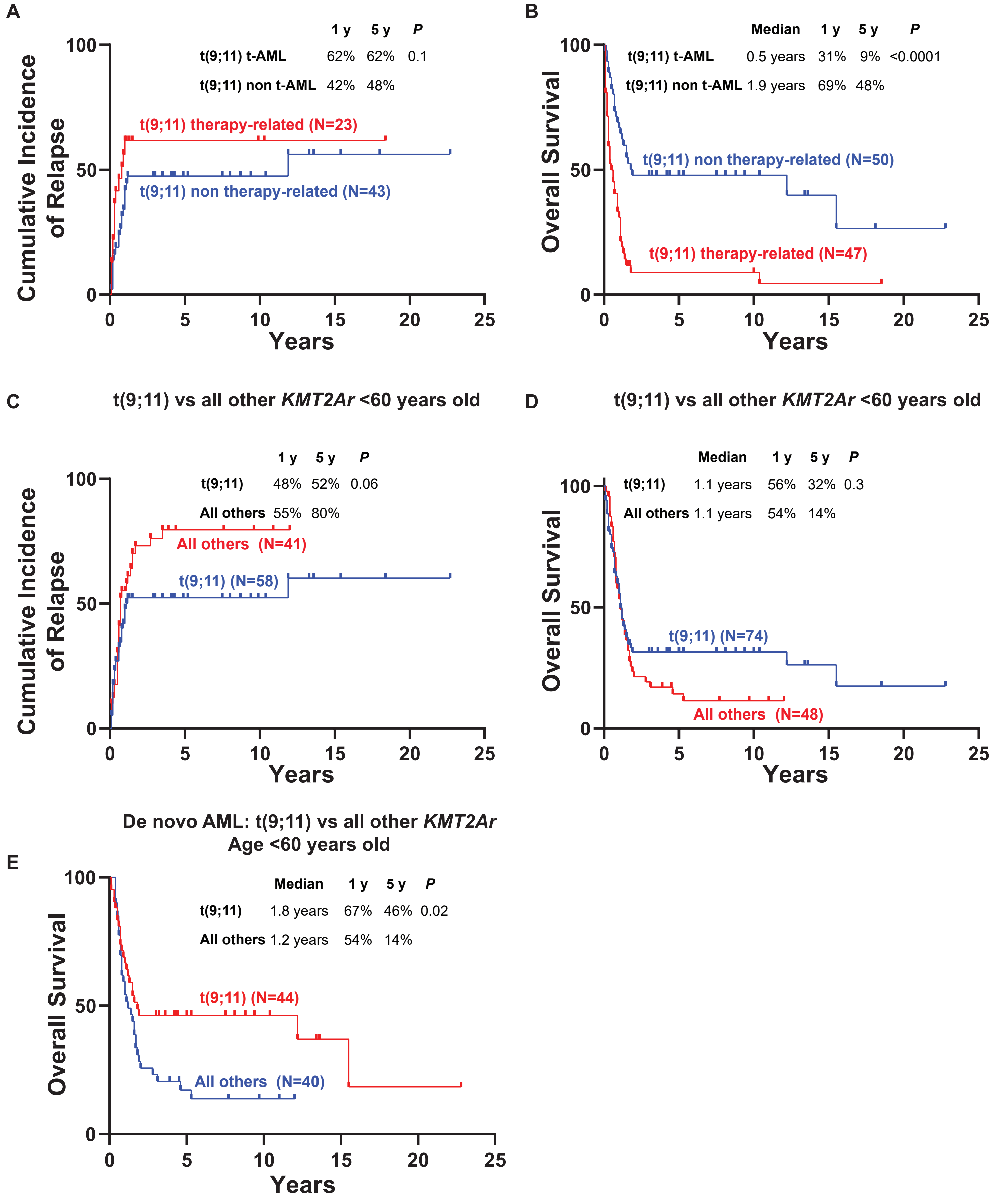

Supplemental Figure 5: Risk of relapse of patients with therapy related *KMT2Ar* AML.

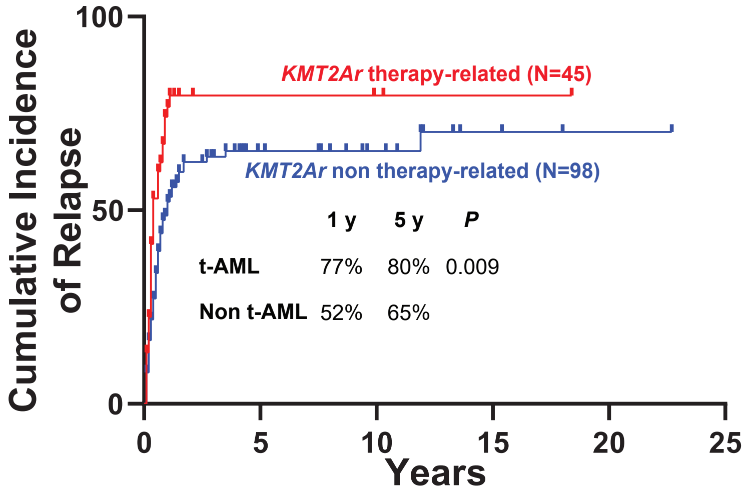

Supplemental Figure 6: Risk of relapse and overall survival of *KMT2Ar* patients with and without additional chromosomal abnormalities (ACA).

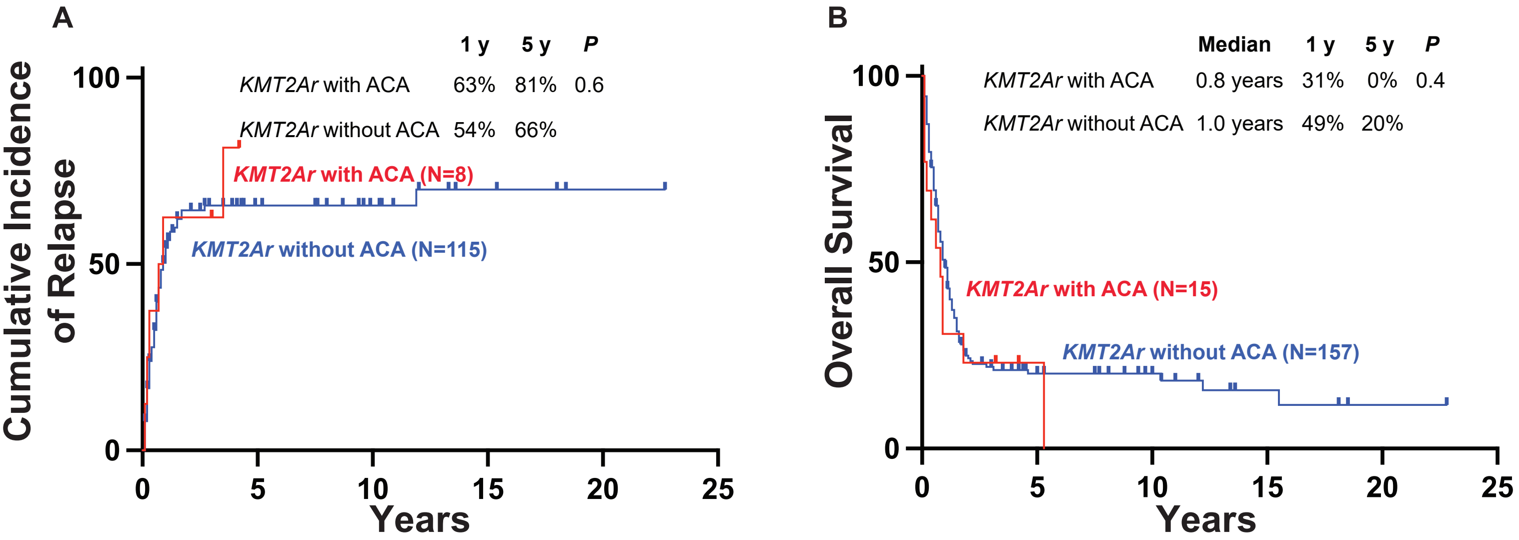

Supplemental Figure 7: Prognostic factors affecting overall survival in *KMT2Ar* AML patients.

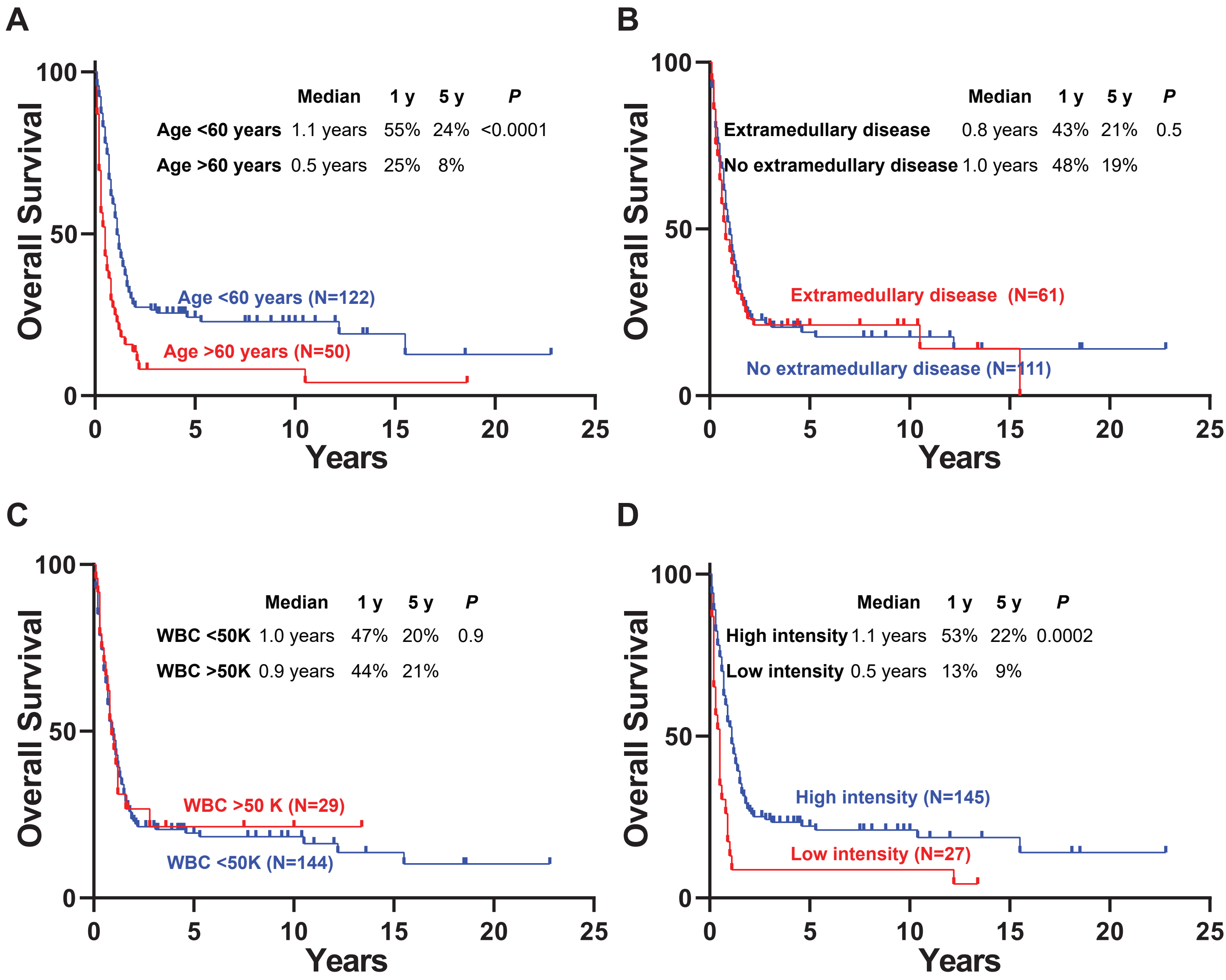

Supplemental Figure 8: Overall survival in *KMT2Ar* AML by mutational status.

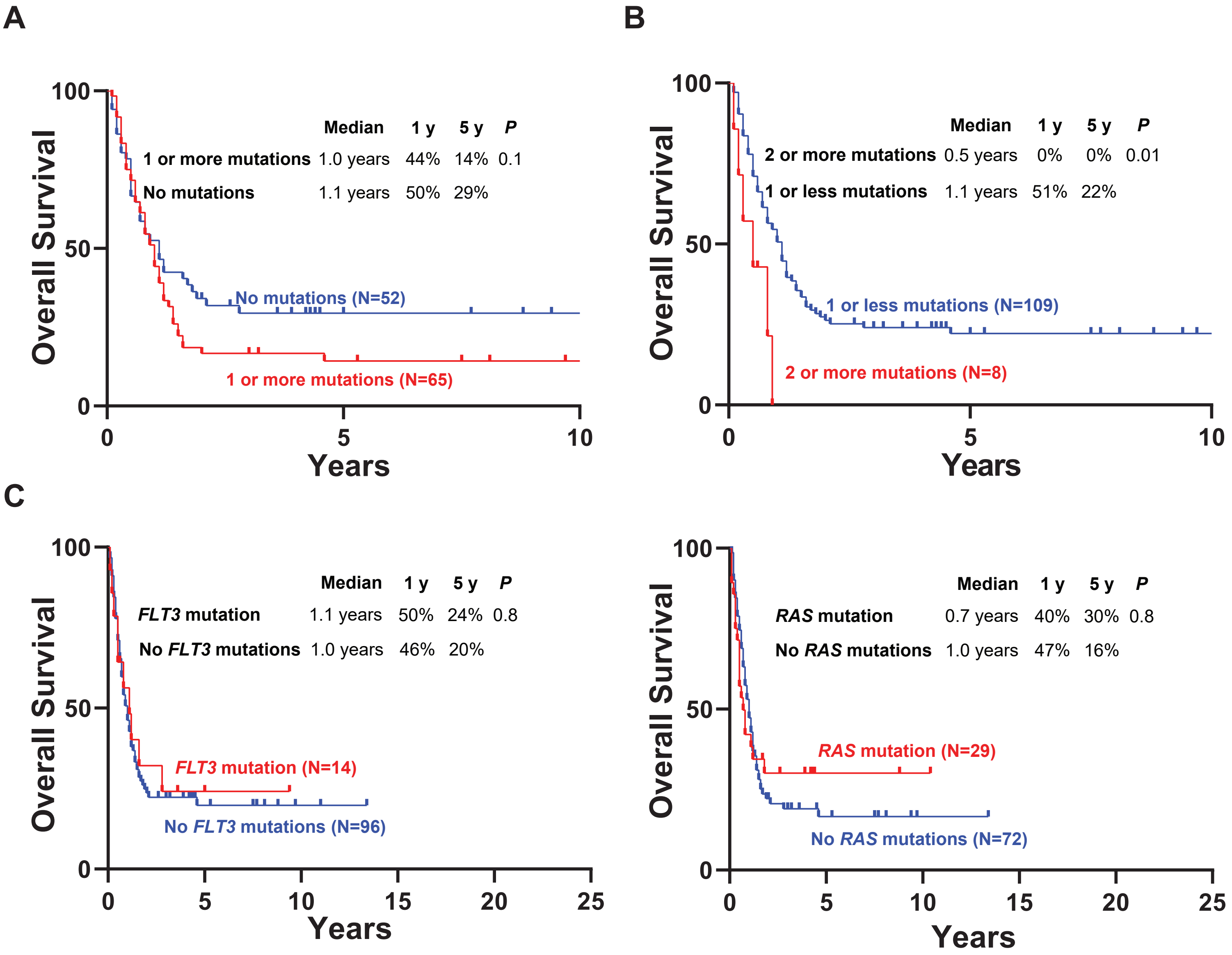

Supplemental Figure 9: Overall survival of adult patients with CNS disease in *KMT2Ar* AML.

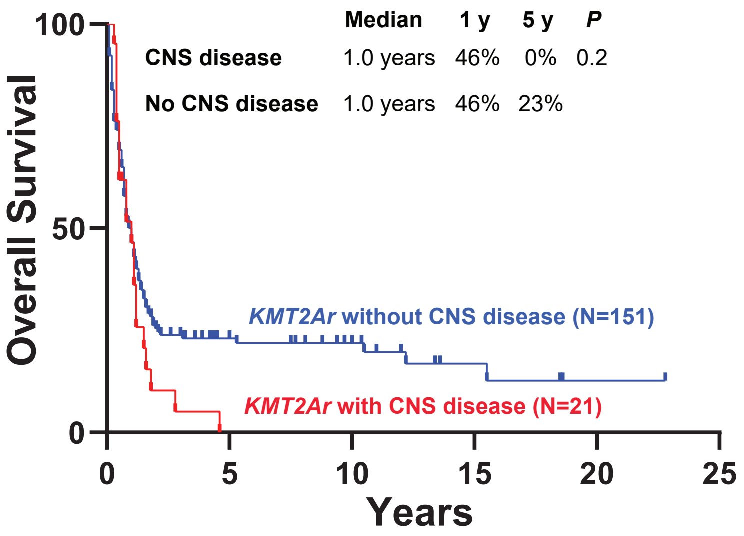

Supplemental Figure 10: Fusion gene partners in relapsed/refractory *KMT2Ar* AML cohort.

A

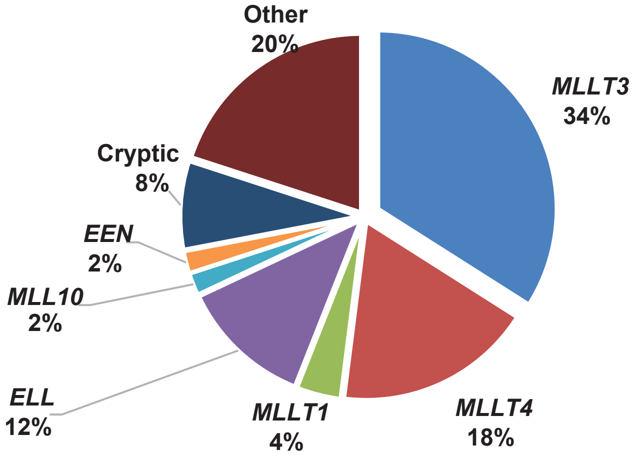

B

Fusion	Cytogenetics	N (50)
KMT2A-MLLT3	t(9;11) (p21;q23)	17 (34)
KMT2A-MLLT4	t(6;11)(q27;q23)	9 (18)
KMT2A-ELL	t(11;19)(q23;p13.1)	6 (12)
KMT2A-MLLT1	t(11;19)(q23;p13.3)	2 (4)
KMT2A-EEN	t(11;19)(q23;p13)	1 (2)
KMT2A-MLLT10	t(10;11)(p12;q23)	1 (2)
Cryptic		4 (8)
Other		10 (20)

Values are N (%).

Supplemental Table 1: Panels of 28, 53, or 81 genes.

Gene	Exons (codons) tested on 81-gene panel	Exons (codons) tested on 53-gene panel	Exons (codons) tested on 28-gene panel
ANKRD26	(NM_014915) 1 (1-6)	(NM_007313): 4-6 (243-362), 7 (395-424)	(NM_005157): 1-11 (1-1004), 11 (1079-1150)
ABL1		(NM_007313): 4-6 (243-362), 7 (395-424)	(NM_005157): 1-11 (1-1004), 11 (1079-1150)
AKT1		(NM_005163): 3 (16-49)	
ALK		(NM_004304): 23 (1172-1175), 25 (1248-1275)	
APC		(NM_000038): 16 (875-918), 16 (1113-1153), 16 (1257-1297), 16 (1288-1328), 16 (1318-1357), 16 (1349-1386), 16 (1377-1575	
ASXL1	(NM_015338): 11-12 (362-1289), 12 (1299-1443), 12 (1450-1542)		(NM_015338): 2-12 (20-1542)
ASXL2	(NM_018263): 11-12 (381-1436)		
ATM		(NM_000051): 8 (353-355), 9 (409-412), 12 (601-633), 17 (846-880), 26 (1308-1331), 34 (1678-1719), 35 (1741-1773), 36 (1792-1832), 39 (1940-1973), 50 (2441-2479), 54 (2665-2670), 55 (2694-2717), 56 (2725-2756), 59 (2889-2891), 61 (2946-2950), 63 (3007-3051)	
BRAF	(NM_004333) 11 (439-478), 15 (581-620)	(NM_004333): 11 (439-471), 15 (581-606)	(NM_004333): 1 (1-23), 1-18 (34-717), 18 (725-767)
BCOR	(NM_017745): 2-3 (1-55), 4 (642-999), 4 (514-635), 4 (460-510), 4 (56-321), 4 (331-454), 5-6 (1000-1080), 7 (1137-1138), 8 (1168-1206), 8(1212-1249), 9 (1275-1340), 9-10 (1354-1366), 10-12 (1401-1547), 13-15 (1556-1640), 15 (1663-1722)		
BCORL1	(NM_021946) 1-3 (1-113), 3 (123-609), 3 (613-808), 3 (832-850), 3-4 (862-1203), 5-6 (1205-1261), 6 (1292-1323), 6-7 (1326-1378), 7(1382-1428), 7-8 (1431-1484), 9-11 (1491-1600), 11-12 (1606-1684), 12 (1687-1699), 12 (1707-1712)		
BRINP3	(NM_199051) 2-8 (1-767)		
CDH1		(NM_004360): 3 (77-117), 8 (369-379), 9 (399-439)	
CALR	(NM_004343) 9 (352-359), 9 (364-410), 9 (417)		
CSF1R		(NM_005211): 7 (297-301), 22 (926-970)	
CSF3R	(NM_156039) 14 (575-622), 17 (681-800), 17 (822-864)		
CBL	(NM_005188) 7-9 (336-477)		
CBLB	(NM_170662) 7-9 (282-397), 10 (402-469)		
CBLC	(NM_012116) 7-9 (336-454), 10 (466-475)		

CEBPA	(NM_004364) 1 (1-70), 1 (249-358), 1 (146-175), 1 (178-201)		
CTNNB1		(NM_001904): 3 (12-50)	
CREBBP	(NM_004380) 1 (1-26), 2-7 (29-559), 8 (561-608), 9-11 (615-720), 12-14 (722-837), 14-30 (840-1724), 31 (2238-2443), 31 (2051-2235), 31(2049), 31 (2011-2037), 31 (1725-1931), 31 (1967-1995)		
CUX1	(NM_181552) 2-6 (11-172), 6-9 (174-241), 10-13 (248-375), 14 (379-408)		
CRLF2	(NM_022148.2) exon 6 (codons 217-256)		
DNMT3A	(NM_022552) 8-22 (286-862), 23 (866-913)	(NM_022552): 23 (866-913)	(NM_022552): 2-23 (1-913)
DDX41	(NM_016222) 1-11 (1-410), 12-14 (420-517), 15 (522-541), 16-17 (546-623)		
EED	(NM_003797) 1-2 (1-69), 2-8 (71-287), 9-12 (289-442)		
ELANE	(NM_001972) 1-2 (5-46), 2 (69-75), 4-5 (123-268)		
ETNK1	(NM_018638) 3 (228-275)		
ETV6	(NM_001987) 1-6 (1-378), 7-8 (385-453)		
EGFR		(NM_005228): 3 (108-142), 7 (288-297), 15 (598-627), 18-20 (708-817), 21 (857-875)	(NM_005228): 1 (1-24), 2-20 (30-786), 20-23 (792-927), 23-26 (935-1043), 26-27 (1047-1061), 27-28 (1067-1211)
ERBB2		(NM_004448): 19 (754-769), 20 (772-818), 21 (839-883)	
ERBB4		(NM_005235): 3 (98-140), 4 (153-186), 6 (208-244), 7 (248-287), 8 (295-306), 9 (333-350), 15 (579-619), 23 (907-936)	
EZH2	(NM_004456) 2-5 (1-158), 5-6 (160-205), 7 (209-217), 8-19 (243-732), 20 (752)	(NM_004456): 16 (618-649)	(NM_004456): 2-5 (3-162), 6-7 (171-217), 8-20 (243-752)
FBXW7	(NM_033632) 9-12 (413-708)	(NM_033632):5 (243-278), 8 (375-394), 9 (429-471), 10 (473-508), 11 (549-583)	
FGFR1		(NM_015850): 4 (120-126)	
FGFR2		(NM_000141): 7 (250-311), 7 (302-313), 9 (362-382), 12 (521-550)	
FGFR3		(NM_000142): 7 (247-288), 9 (379-422), 14-15 (639-659), 18 (792-807)	
FLT3	(NM_004119) 11-20 (437-847)	(NM_004119): 11 (437-456), 14 (569-605), 16 (648-683), 20 (807-843)	(NM_004119): 2-24 (15-994)
GATA1	(NM_002049) 2-3 (1-84)		(NM_002049): 2-5 (1-287), 6 (291-405), 6 (407-414)
GATA2	(NM_032638) 2-5 (22-377), 5-6 (379-481)		(NM_032638): 2-6 (1-481)
GFI1	(NM_005263) 2 (2-39)		
GNA11		(NM_002067): 4-5 (172-216), 6 (255-297)	
GNAQ		(NM_002072): 4-5 (159-245), 5-6 (241-297), 6-7 (291-360), 7 (355-360)	
GNAS	(NM_000516) 8 (200-202), 11 (315-324)	(NM_000516): 8 (200-220)	
HNRNPK	(NM_002140) 3-17 (1-465)		

HNF1A		(NM_000545): 3 (205-238), 4 (271-314)	
HRAS	(NM_005343) 2-3 (1-59), 3-4 (87-135), 4 (137-150)	(NM_005343): 2 (1-15), 3 (38-63)	(NM_005343): 2-5 (1-190)
IDH1	(NM_005896) 4 (132-133)	(NM_005896): 3-10 (1-415)	(NM_005896): 3-10 (1-415)
IDH2	NM_002168) 4 (125-178)	(NM_002168): 4 (125-178)	(NM_002168): 1 (1-14), 1-11 (26-453)
IKZF2	(NM_006060) 2-8 (1-377), 8 (383-392), 8 (403-430), 8 (445), 8 (481-493)		(NM_016260): 2-8 (1-527)
IL2RG	(NM_000206) 1-2 (1-45), 2-4 (51-195), 5-8 (199-339)		
IL7R	(NM_002185) 5-7 (180-292)		
JAK1	(NM_002227) 3-9 (3-445), 10 (447-465), 10-22 (470-1023), 22-24 (1026-1123)		
JAK2	(NM_004972) 10 (405-442), 12-14 (505-622), 16 (665-711), 18 (762-812)	(NM_004972): 14 (615-622)	(NM_004972): 3-18 (1-806), 19-25 (812-1133)
JAK3	(NM_000215) 2-6 (1-284), 7-13 (288-590), 14-23 (596-1069)	(NM_000215): 13 (568-573), 16 (683-723)	
KDM6A	(NM_021140) 2-9 (54-223), 9-16 (228-631), 17-19 (642-971), 20-21 (980-1070), 22-29 (1080-1402)		
KDR		(NM_002253); 6 (220-248), 7 (267-276), 11 (471-476), 19 (872-874), 21 (946-985), 26 (1135-1146), 27 (1171-1211), 30 (1308-1357)	
KIT	(NM_000222) 8-9 (411-514), 11 (550-592), 17 (788-828)	(NM_000222): 2 (51-93), 9-10 (502-547), 10-11 (540-592), 13 (641-664), 14 (670-712), 15 (714-745), 17 (815-828), 18 (838-866)	(NM_000222): 1-6 (1-334), 6-21 (367-977)
KLHL6		(NM_130446): 1 (1-98)	
KRAS	(NM_004985) 2-4 (1-150)	(NM_004985): 2 (1-22), 3 (38-63), 4 (103-147)	(NM_004985): 2-5 (1-189)
MAP2K1	(NM_002755) 2 (27-90), 3 (98-146)		
MDM2			(NM_002392): 1-11 (1-498)
MET		(NM_001127500): 2 (168-209), 2 (375-400), 14 (1008-1028), 16 (1110-1132), 19 (1247-1284)	
MLH1		(NM_000249): 12 (383-426)	
MLL(KMT2A)	(NM_005933) 2 (145-168), 3-4 (176-1075), 4 (1081-1112), 5 (1117-1184), 6 (1190-1212), 7 (1224-1325), 8-13 (1338-1560), 14-15 (1566-1665), 27 (2186-2195), 27 (2201-2355), 27 (2373-2452), 27 (2472-3215), 27 (3223-3324), 27 (3339-3575)		(NM_005933): 1-5 (103-1190), 6-36 (1207-3970)
MPL	(NM_005373):10 (490-522), 12 (552-636)	(NM_005373): 10 (514-522)	(NM_005373): 1-9 (1-488), 10-12 (490-636)
MYD88		(NM_002468): 1 (1-6), 1-5 (15-310)	
NF1	(NM_001042492) 2-5 (21-189), 6 (201-218), 8-13 (244-467), 13-17 (478-667), 18 (674-728), 18-22 (746-992), 23-24 (997-1066), 25-26 (1082-1146), 26-31 (1160-1378), 31-35 (1382-1549),		

	35-38 (1564-1868), 39 (1870-1884), 39-47 (1886-2322), 47-52 (2325-2555), 52-58 (2568-2840)		
NOTCH1	(NM_017617) 26 (1529-1594), 26-28 (1602-1795), 34 (2550-2556), 34 (2290-2543), 34 (2069-2229), 34 (2234-2273), 34 (2550-2556), 34(2290-2543), 34 (2069-2229), 34 (2234-2273)	(NM_017617): 26 (1562-1601), 27 (1673-1679)	(NM_017617): 2-31 (21-1893), 31-34 (1902-2286), 34 (2289-2552)
NPM1	(NM_002520) 11 (283-295)	(NM_002520): 11 (283-295)	(NM_002520): 1-7 (1-181), 7-11 (187-295)
NRAS	(NM_002524) 2-4 (1-150)	(NM_002524): 2 (1-18), 3 (38-62)	(NM_002524): 2-5 (1-190)
PAX5	(NM_016734) 1-10 (14-392)		
PDGFRA		(NM_006206): 12 (552-592), 14 (659-668), 15 (673-717), 18 (823-854)	
PHF6	(NM_032458) 2-3 (1-78), 4-10 (81-366)		
PIGA	(NM_002641) 2 (1-6), 2-5 (16-396), 6 (399-418), 6 (422-485)		
PML	(NM_033238) 3 (201-255)		
PRPF40B	(NM_001031698) 2-19 (2-609), 19 (611-626), 20 (629-658), 20-26 (661-893)		
PIK3CA		(NM_006218): 2 (83-118), 5 (345-353), 8 (418-445), 10 (538-555), 14 (701-729), 21 (988-1069)	
PTPN11	(NM_002834) 3-4 (46-125), 7 (253-285), 12 (460-462), 12-13 (465-533)	(NM_002834): 3 (59-104), 13 (501-533)	(NM_002834): 1-4 (1-157), 5-15 (176-594)
PTEN	(NM_000314) 7-8 (212-285), 8 (290-342)	(NM_000314): 1 (5-27), 3 (67-70), 6 (170-210), 7 (212-266), 8 (287-342)	
RAD21	(NM_006265) 2-13 (1-560), 14 (569-632)		
RARA	(NM_000964) 6-7 (211-338)		
RB1		(NM_000321): 4 (127-158), 6 (199-203), 11 (357-376), 18 (570-605), 20 (659-700), 21 (703-733), 22 (746-775)	
RET		(NM_020975): 10-11 (610-667), 13 (766-798), 15 (880-910), 16 (918-934)	
RUNX1	(NM_001754) 2-4 (1-70), 4-9 (79-437)		(NM_001754): 2-4 (1-70), 4-9 (73-437)
SETBP1	(NM_015559) 4 (838-885)		
SF1	(NM_004630) 2-12 (11-524), 13 (528-578), 13 (582-640)		
SF3A1	(NM_005877) 1-7 (1-322), 7-9 (329-424), 9-12 (427-634), 13-16 (651-794)		
SF3B1	(NM_012433) 13-16 (574-790)		
SH2B3	(NM_005475) 2 (1-39), 2 (43-99), 2 (132-150), 2 (158-164), 2-8 (212-576)		
SMC1A	(NM_006306) 1-5 (13-283), 6-7 (285-415), 8-14 (419-771), 15-19 (775-976), 20-22 (992-1146), 23-24		

	(1148-1206), 25 (1217-1234)		
SMC3	(NM_005445) 1 (1-5), 2-6 (19-110), 6-16 (113-504), 16-17 (507-580), 17-25 (591-975), 25-29 (979-1217)		
SRSF2	(NM_003016) 1 (1-38), 1 (45-121)		
STAG1	(NM_005862) 2 (1-5), 3-20 (10-703), 21-22 (718-738), 22-27 (740-953), 27-34 (955-1259)		
STAG2	(NM_006603) 2-15 (1-512), 16 (521-534), 16-21 (541-711), 21-33 (714-1232)		
STAT3	(NM_139276) 17 (489-503), 18-22 (534-715)		
SMAD4		(NM_005359): 3 (119-142), 5 (167-208), 6 (243-263), 8 (310-319), 9 (329-373), 10 (385-424), 11 (443-480), 12 (496-535)	
SMARCB1		(NM_003073): 2 (39-78), 4 (156-167), 5 (199-210)	
STAT5A	(NM_003152) 3-6 (1-177), 6-7 (181-206), 8-9 (266-286), 9-20 (315-795)		
STAT5B	(NM_012448) 16 (636-693)		
SMO		(NM_005631): 3 (197-242), 5 (323-366), 6 (403-422), 9 (533-551)	
SUZ12	(NM_015355) 1 (20-44), 1 (46-84), 2 (92-107), 4-5 (129-169), 7-16 (198-740)		
SRC		(NM_005417): 14 (530-537)	
STK11		(NM_000455): (36-77), 4-5 (193-211), 6 (261-288), 8 (332-370)	
TET2	(NM_001127208) 3 (1-77), 3 (91-826), 3 (829-853), 3-11 (867-2003) (NM_000546) 2 (1-25), 4 (80-125), 5-11 (134-394)		(NM_001127208): 3 (1-854), 3-11 (866-2003)
TP53	(NM_000546) 2 (1-25), 4 (80-125), 5 (134-187), 6-11 (190-394)	(NM_000546): 2 (1-12), 4 (69-112), 5 (126-186), 5-6 (181-192), 6 (187-223), 6-7 (214-253), 8 (267-306), 10 (332-342)	(NM_000546): 2-11 (1-394)
TERC	81 gene panel		
TERT	(NM_198253) 1 (1-24), 2 (349-525), 2 (80-164), 2 (258-342), 3-4 (525-630), 4-5 (633-663), 5 (675-677), 6 (711-749), 6-8 (753-800), 8-11 (805-925), 11-16 (933-1133)		
U2AF1	(NM_006758) 2 (15-44), 6 (117-161)		
U2AF2	(NM_007279) 1 (1-17), 3-5 (62-161), 6-12 (163-437), 12 (441-473)		
VHL		(NM_000551): (88-114), 2 (129-155), 3 (157-200)	
WT1	(NM_024426) 1 (187-216), 1 (179-184), 1 (2-44), 1 (122-165), 2-10 (216-518)		(NM_024426): 1 (1-59, 72-105, 122-216), 2-10 (216-518)
XPO1		(NM_003400) 14-15 (501-575)	

ZRSR2	(NM_005089) 1-3 (1-68), 4 (71-90), 6-8 (134-201), 8 (212-257), 10-11 (276-417), 11 (476-483)	

Supplemental Table 2: Baseline characteristics of relapsed/refractory adult AML with *KMT2Ar*.

Characteristic	*KMT2Ar*	Age-matched diploid	*P*
**Patients, no.**	50	177	
**Median age, years (range)**	47 (22-75)	54 (10-85)	0.07
**Female, no. (%)**	28 (56)	79 (45)	0.2
**Monocytic phenotype, no. (%)**	15 (30)	44 (25)	0.5
**WBC, median x 10^9/L (range)**	4.3 (0-135)	8 (0-250)	0.03
**Platelets, median x 10^9/L (range)**	54 (7-358)	56 (1-497)	0.8
**BM blast %, median (range)**	51 (0-94)	48 (15-96)	0.4
**High intensity treatment, no. (%)**	46 (92)	138 (78)	0.03
**Low intensity treatment, no. (%)**	4 (8)	39 (22)	
**Allo-HSCT, no. (%)**	18 (36)	34 (19)	0.02

High intensity treatment includes treatment with the combination of cytarabine and idarubicin or the addition of a nucleoside analog to the combination. Low intensity treatment includes treatment with hypomethylating agents, low-dose cytarabine, or targeted therapies.
WBC, white blood cell; BM, bone marrow; LDH, lactate dehydrogenase; Allo-HSCT, allogeneic hematopoietic stem cell transplant; t-aml; therapy related AML.
P: Kruskal-Wallis or Fisher exact test.

Supplemental Table 3: Karyotype of 10 patients with less common *KMT2A* rearrangements who constitute the "Other" group in relapse/refractory cohort.

Age and Sex	Karyotype
31/F	(12)46XX,3q-,t(11q;15q);(9)45-46",+other changes;(4)+6
43/F	"47,XX,t(3;7)(p21;p22),t(4;10;15)(q21;p13;q15),+6,add(6)(q21),del(9)(q13q22),t(11;22)(q23;q11.2)[13]
58/F	45,XX,t(5;11)(q35;q23),-10,ider(17)(q10)del(17)(q11.2q21)[11]; 46,XX[9]
75/M	47,XY,t(3;11)(q12;q23),+8[2]; 47,idem,del(2)(q33)[18]
50/F	46,XX,t(8;11)(q13;p12)[1];46,XX[19]
5 patients	Unknown karyotype

Supplemental Table 4: Treatments for newly diagnosed *KMT2Ar* AML patients.

Treatment regimen	N (%)
**High intensity**	145 (84)
IA (Idarubicin + Cytarabine)	60 (41)
CLIA (Cladribine + IA)	9 (6)
FIA (Fludarabine + IA)	11 (8)
CIA (Clofarabine + IA)	17 (12)
FLAG-Ida + venetoclax	2 (1)
CLIA + venetoclax	3 (2)
Other	43 (30)
**Low intensity**	27 (16)
LDAC	2 (7)
HMA	9 (33)
HMA + venetoclax	5 (19)
Other	11 (41)

LDAC, low-dose cytarabine; HMA, hypomethylating agents.
Midostaurin was added to therapy in 3 of 14 patients with *KMT2Ar* and *FLT3* mutations.
Other treatments include investigational agents as single agents or in combinations with high intensity chemotherapy or less commonly used regimens.

Supplemental Table 5: Karyotype of 17 patients with less common *KMT2A* rearrangements who constitute the "Other" group.

Age and Sex	Karyotype
27/M	(25)46XY,t(11q-;17q+)(q23;q25)
31/M	(17)46XY,t(10p-;11q+);(7)46XY,1p+,t(10p-;11q+);(1)46XY
51/F	(19)47XX,t(7;11)(q11.2;q23),+8;(2)DIP
21/M	t(x;11)(q22;q23)
48/F	47,XY,t(3;11)(q12;q23),+8[2]; 47,idem,del(2)(q33)[18]
51/F	t(11;22)(q23;q12)
40/F	46XX,DER(2)t(2;11)(q31;q23)[20]
73/M	t(1;11)(p32;q23)
54/F	t(11;17)(q23;q25)
64/F	t(2;11)(p21;q23),
31/F	t(7;11)(q32;q23)
50/F	t(1;11)(p34;q13),
70/M	t(11;16)(q23;p13.3),
32/M	t(11;17)(q23;q12),
50/M	t(11;17)(q23;q25)
71/M	t(11;15)(q23;q14),
55/M	t(11;17)(q23;q12),

Supplemental Table 6: Baseline characteristics of newly diagnosed *KMT2Ar* AML by subgroups.

Characteristic	t(9;11)	t(6;11)	*P*	t(11;19)	*P*	t(11;v)(q23;v)	*P*
**Patients, no.**	97	19		34		22	
**Median age, years (range)**	52 (17-82)	54 (22-85)	0.8	52 (23-83)	0.7	52 (20-73)	0.9
**Female, no. (%)**	65 (67)	11 (58)	0.4	18 (53)	0.2	10 (45)	0.09
**Monocytic phenotype, no. (%)**	73 (75)	14 (74)	0.9	19 (56)	**0.048**	10 (45)	**0.009**
**WBC, median x 10^9/L (range)**	9 (1-270)	9 (1-134)	0.9	9 (1-111)	0.9	9 (1-106)	0.9
**Platelets, median x 10^9/L (range)**	48 (4-235)	49 (9-155)	0.9	49 (10-187)	0.9	49 (3-279)	0.9
**BM blast %, median (range)**	76 (20-98)	76 (22-94)	0.9	75 (22-92)	0.9	76 (21-94)	0.9
**t-AML, no. (%)**	47 (48)	5 (26)	0.08	10 (29)	0.07	7 (32)	0.2
**High intensity treatment, no. (%)**	82 (85)	17 (89)	0.7	26 (76)	0.3	20 (91)	0.7
**Low intensity treatment, no. (%)**	15 (15)	2 (11)		8 (24)		2 (9)	
**Allo-HSCT, no. (%)**	26 (27)	8 (42)	0.3	7 (21)	0.6	5 (23)	0.8

High intensity treatment includes treatment with the combination of cytarabine and idarubicin or the addition of a nucleoside analog to the combination. Low intensity treatment includes treatment with hypomethylating agents, low-dose cytarabine, or targeted therapies.

WBC, white blood cell; BM, bone marrow; LDH, lactate dehydrogenase; Allo-HSCT, allogeneic hematopoietic stem cell transplant; t-aml; therapy related AML.

P: Kruskal-Wallis or Fisher exact test.

Supplemental Table 7: Frequency of mutations by 11q23 translocation.

	t(9;11)	t(6;11)	*P*	t(11;19)	*P*	t(11;v)(q23;v)	*P*
RAS	16/52 (31)	5/15 (33)	0.9	7/21 (33)	0.9	1/15 (7)	0.09
FLT3	8/55 (15)	3/16 (19)	0.7	2/22 (9)	0.7	1/17 (6)	0.7
PTPN11	1/20 (5)	0/3 (0)	0.9	1/9 (11)	0.5	0/7 (0)	0.9
TP53	0/22 (0)	1/5 (20)	0.2	0/9 (0)	0.9	1/7 (14)	0.2
CEBPA	1/29 (3)	0/7 (0)	0.9	1/13 (8)	0.5	0/8 (0)	0.9
IDH 1/2	2/30 (7)	0/10 (0)	0.9	0/14 (0)	0.9	0/8 (0)	0.9
WT1	0/17 (0)	0/2 (0)	0.9	1/14 (7)	0.5	0/4 (0)	0.9

Values are mutated/tested (%). Subgroups were compared to t(9;11).

Supplemental Table 8: Responses of newly diagnosed *KMT2Ar* AML by subgroups.

Best Response	t(9;11)	t(6;11)	*P*	t(11;19)	*P*	t(11;v)(q23;v)	*P*
**N**	97	19		34		22	
**CR**	63 (65)	11 (58)	0.9	23 (68)	0.7	16 (73)	0.1
**CRi**	3 (3)	2 (11)		2 (6)		3 (14)	
**CR+CRi**	66 (68)	13 (68)		25 (74)		19 (86)	
**No response**	31 (32)	6 (32)		9 (27)		3 (14)	
**MRD negative by MFC**	10/11 (91)	1/2 (50)	0.3	3/7 (43)	0.047	3/5 (60)	0.2

Values are n (%).
CR, complete remission; CRi, complete remission with incomplete hematologic recovery; MRD, minimal residual disease assessed by multiparameter flow cytometry (MFC) following induction in evaluable patients.
P: Kruskal-Wallis or Fisher exact test. All subgroups were compared to t(9;11).

Supplemental Table 9: Responses by lines of therapy for adults with AML.

	1st Line			2nd Line			≥ 3rd Line		
	KMT2Ar	**Diploid**	***P***	***KMT2Ar***	**Diploid**	***P***	***KMT2Ar***	**Diploid**	***P***
**N**	172	522		91	178		217	112	
**CR**	113 (66)	403 (77)	0.01	31 (34)	61 (34)	0.8	11 (5)	17 (15)	< 0.001
**CRi**	10 (6)	18 (4)		8 (9)	19 (11)		9 (4)	18 (16)	
**CR+CRi**	123 (72)	421 (81)		39 (43)	80 (45)		20 (9)	35 (31)	
**No response**	49 (28)	101 (19)		52 (57)	98 (55)		197 (91)	77 (69)	
**MRD negative by MFC**	17/25 (68)	90/132 (68)	0.9	12/16 (75)	16/24 (67)	0.7	1/3 (33)	8/9 (89)	0.1

Values are n (%).
CR, complete remission; CRi, complete remission with incomplete hematologic recovery; MRD, minimal residual disease assessed by multiparameter flow cytometry (MFC) following induction in evaluable patients.

Supplemental Table 10: Variables associated with CNS disease in adults with KMT2Ar AML.

Variables	CNS +	CNS-	*P*
**Patients, no. (%)**	20 (12)	152 (88)	
**WBC, median x 10^9/L (range)**	8.5 (1-108)	9.1 (1-270)	0.9
**Platelets, median x 10^9/L (range)**	49 (21-155)	48 (3-279)	0.9
**BM blast %, median (range)**	76 (38-94)	76 (20-98)	0.9
**t-AML, no. (%)**	5 (25)	64 (42)	0.2
***RAS*, no. (%)**	5/18 (28)	24/85 (28)	0.9
***FLT3*, no. (%)**	4/18 (22)	10/92 (11)	0.2
***TP53*, no. (%)**	0/6 (0)	2/37 (5)	0.9
**≥1 mutations, no. (%)**	8/18 (44)	44/99 (44)	0.9
**≥2 mutations, no. (%)**	1/18 (6)	7/99 (7)	0.9
**t(9;11), no. (%)**	8 (40)	89 (59)	0.2
**t(6;11), no. (%)**	3 (15)	16 (11)	0.5
**t(11;19), no. (%)**	5 (24)	29 (19)	0.6
**t(11;v)(q23;v), no. (%)**	5 (25)	29 (19)	0.6
**Extra-medullary disease, no. (%)**	7 (35)	54 (36)	0.9
**Monocytic phenotype, no. (%)**	14 (70)	102 (67)	0.9

Values are n (%).
WBC white blood cell; BM, bone marrow; LDH, lactate dehydrogenase; t-aml; therapy related AML; values indicate mutated/tested genes. Extramedullary disease at diagnosis.
P: Kruskal-Wallis or Fisher exact test.
